# HeatMapper: powerful combined visualization of gene expression profile correlations, genotypes, phenotypes and sample characteristics

**DOI:** 10.1186/1471-2105-7-337

**Published:** 2006-07-12

**Authors:** Roel GW Verhaak, Mathijs A Sanders, Maarten A Bijl, Ruud Delwel, Sebastiaan Horsman, Michael J Moorhouse, Peter J van der Spek, Bob Löwenberg, Peter JM Valk

**Affiliations:** 1 Department of Hematology, Erasmus University Medical Center, Rotterdam, The Netherlands; 2 Department of Bioinformatics, Erasmus University Medical Center, Rotterdam, The Netherlands

## Abstract

**Background:**

Accurate interpretation of data obtained by unsupervised analysis of large scale expression profiling studies is currently frequently performed by visually combining sample-gene heatmaps and sample characteristics. This method is not optimal for comparing individual samples or groups of samples. Here, we describe an approach to visually integrate the results of unsupervised and supervised cluster analysis using a correlation plot and additional sample metadata.

**Results:**

We have developed a tool called the HeatMapper that provides such visualizations in a dynamic and flexible manner and is available from .

**Conclusion:**

The HeatMapper allows an accessible and comprehensive visualization of the results of gene expression profiling and cluster analysis.

## Background

Gene expression profiling by applying microarrays followed by cluster analyses is a powerful way to define pathobiologically relevant relations between the expression of sets of genes and disease classes. Unsupervised methods such as cluster analysis [1] and principal component analysis [2] are often applied to calculate and visualize these relations. Interpretation of results obtained by cluster analysis is frequently performed by visual inspection of a so-called heatmap; a matrix of genes versus samples in which gene expression levels or ratios are indicated using colors. Green often indicates low expression or down-regulation while red is frequently used to indicate high expression or up-regulation of genes [1,3]. A dendrogram, which is typically produced by unsupervised cluster analysis, provides further insights into sample-to-sample or gene-to-gene relations [1]. These visualizations are useful when small numbers of samples and genes are analyzed, but are insufficient when studying larger datasets. Similarities and differences between samples or genes are easily lost due to the large size of these visualizations. This shortcoming particularly affects patient-cohort studies, since these analyses include increasing numbers of samples to allow comprehensive analyses.

A second type of heatmap that is frequently used is a matrix of pair-wise sample correlations in which anti-correlation or correlation is indicated by a color-scale, e.g. blue to red [4-6]. Although details on individual gene expression measurements are lost, similarity between any pair of samples can easily be inspected.

To be able to correctly interpret both the sample versus gene expression heatmap and the sample versus sample correlation plot, data of the type of samples profiled, e.g. clinical parameters, karyotypes, mutations in particular genes, or gene expression data should be available. This information might then be included in a visual overview, as is frequently seen with sample versus gene heatmaps [7,8]. Such presentation would be a useful addition to the sample-sample heatmaps, which are frequently shown without metadata. Here we developed a tool, called the HeatMapper, which can generate such combined visualizations. The tool is simple in use and allows dynamic and flexible display of a correlation plot in combination with sample characteristics.

## Implementation

The HeatMapper, written in JAVA (version 1.4.2), uses comma-separated or tab-delimited text-files as input. It requires two files: one file containing a matrix of sample-sample similarity, i.e. Pearson correlation, Spearman correlation or Euclidean distance, and one file with sample related data. In both files, similar sample ID's are used. Correlation files can be generated using tools such as Omniviz, GeneMaths and R/BioConductor, while sample data files can for instance be created in Microsoft Excel. Example files are available from the website. Alternatively, the tool can be adapted to communicate with a database. In our laboratory, the HeatMapper is connected to a MySQL database which further optimizes the workflow. This version is available on request.

## Results & discussion

**Figure 1:**
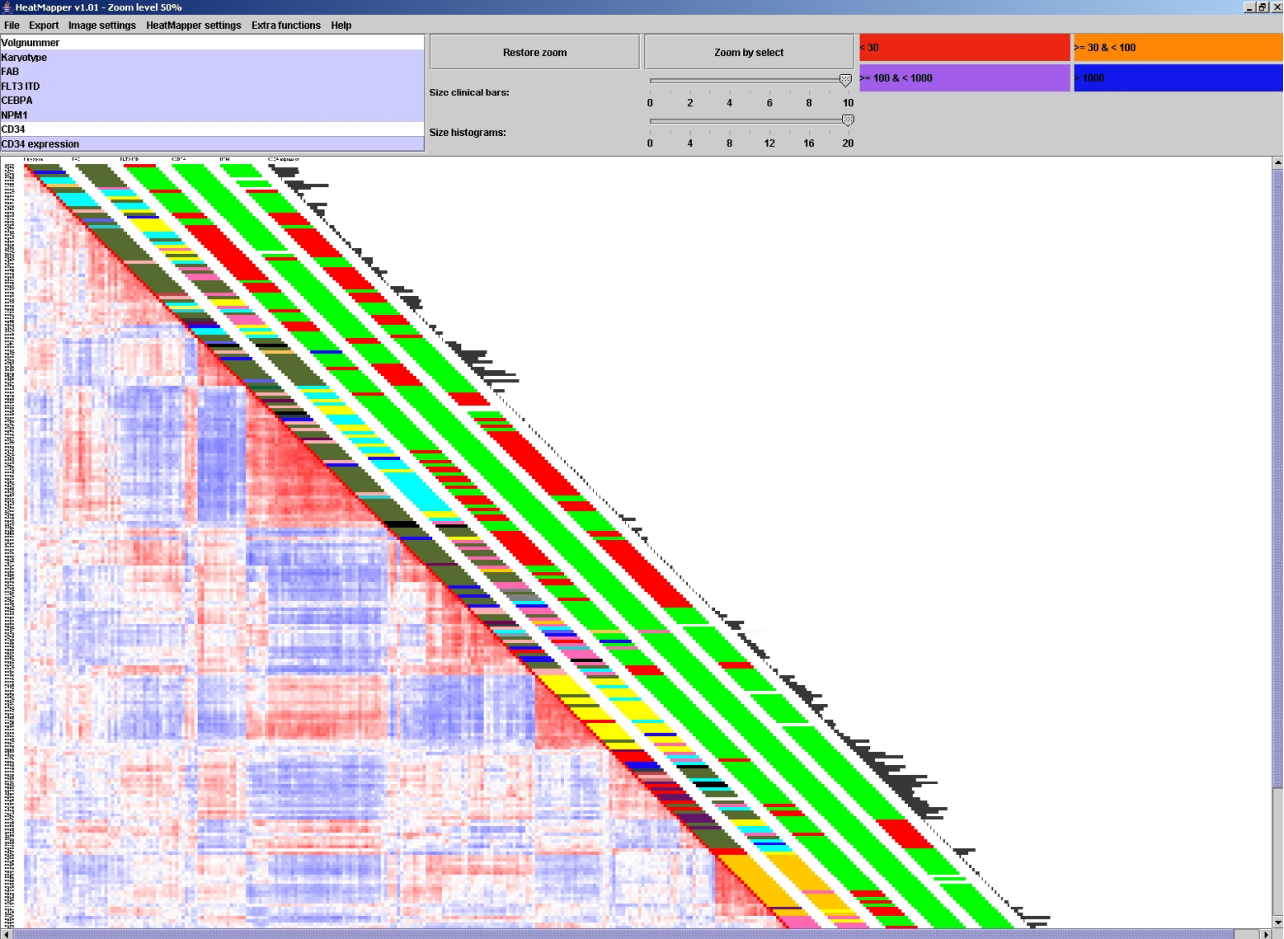
**HeatMapper screenshot**. The figure shows pairwise correlations between 285 samples of patients with Acute Myeloid Leukemia, as described previously [6]. The cells in the visualization are colored by Pearson's correlation coefficient values with deeper colors indicating higher positive (red) or negative (blue) correlations. Clinical and molecular data are depicted in the columns along the original diagonal of the heatmap. Karyotype and FAB classification based on cytogenetics are depicted in the first two columns (karyotype: normal-green, inv(16)-yellow, t(8;21)-purple, t(15;17)-orange, 11q23 abnormalities-blue, 7(q) abnormalities-red, +8-pink, complex-black, other-gray; FAB M0-red, M1-green, M2-purple, M3-orange, M4-yellow, M5-blue, M6-grey). FLT3 ITD, CEBPA and NPM1 mutations are depicted in the same set of columns (red bar: positive and green bar: negative). The expression levels of CD34 (probe set: 209543_s_at) in the 285 AML patients are plotted in the last column (bars are proportional to the level of expression).

As the upper right part of a traditional sample versus sample heatmap is in fact a mirror image of the lower left part, it is redundant. Therefore, when data are loaded, the HeatMapper only displays a triangular heatmap (Figure 1). Sample-sample (dis-) similarity, i.e. Pearson correlation, Spearman correlation or Euclidean distance, is mapped to a color scale ranging from blue to red. Dark blue relates to the negative extreme value of the metric, i.e. -1 for Pearon correlation, where dark red refers to the positive extreme value, i.e. 1 for Pearson correlation. Sample related data, can be simply added via the menu and is subsequently plotted alongside the heatmap diagonal. Different entries in one sample characteristic are mapped to different colors, or, in the case of numeric data, shown as bars of which the size is proportional to the value. Several options are available to customize the resulting visualization, such as zoom functionality and options to change the colors used in histograms or bars to indicate phenotypic or genotypic differences. Further customization options include the possibility to change the sample order, allowing a user for instance to visualize the results of a different clustering algorithm, or to sort the data according to any user-defined order. This can be accomplished via selecting the 'Change sample order' menu-option, after which the order of the sample ids can be inserted by typing them or using copy-paste. Subsets of the original data can be created and viewed in any sequence. Importantly, high-resolution images of the produced figures can be exported using the Portable Network Graphics (PNG) format.

Our tool provides several advantages over more traditional means of presenting results obtained gene expression profiling and clustering analysis [7,8]. The pair-wise display of samples clearly indicates similarity in expression profiles. By combined visualization of sample versus sample similarities and sample characteristics, subclasses of samples sharing a commonality, such as a mutation in a particular gene, and a high similarity in expression profile can be readily identified. Cluster assignments, made manually by the user, can then be added via the 'Add special values' menu option and displayed as sample characteristic.

As an example, Figure 1 shows the results of a cluster analysis of 285 acute myeloid leukemia (AML) samples. Clusters are recognized as red triangles near the plot diagonal. Sample related data are presented in the adjacent bars, where the same color indicates the same characteristic. The last bar indicates the expression levels of CD34, in which the level of expression is proportional to the length of the bar. By visual inspection of this plot, one can immediately conclude that (1)AML samples can be separated into several subtypes, such as cases with a t(8;21), based on expression profiling [9], (2) several clusters are related to a single distinguished abnormality (for instance nucleophosmin (*NPM1*) mutations), indicated in red in the fifth column and (3) mRNA levels of CD34 are low in samples with *NPM1 *mutations.

In our laboratory the HeatMapper code has been coupled to a database containing gene expression profiling results, from which gene expression levels can dynamically be obtained. This allows the quick and accurate visual inspection of the distribution of expression levels in different clusters, and making the tool even more powerful. The database implementation, is available on request.

Our visualization method has been successfully applied in several studies [6, 9, 10, 11, 12].

## Conclusion

With the increase of the number of samples profiled, particularly in patient-cohort studies, specialized visualization methods for microarray studies are indispensable. Our tool allows the accurate inspection of combinations of dataset characteristics, i.e. correlations and clustering results and sample related characteristics, i.e. survival time and gene expression levels. Summarizing, the HeatMapper tool results in powerful visualization tool that allows the accurate and rapid interpretation of the data obtained by large scale gene expression profiling. The HeatMapper tool has already proven to be very useful in several studies [6, 9, 10, 11, 12].

## Availability & requirements

Project name: HeatMapper

Project homepage: 

Operating system: Platform independent

Programming language: JAVA

Other requirements: JAVA 1.4.2 or higher.

License: The tool is available free of charge. Source code is available upon request.

Any restrictions to use by non-academics: None

## Abbreviations

AML Acute Myeloid Leukemia

PNG Portable Network Graphics

NPM1 Nucleophosmin

## Authors' contributions

RGWV designed the software, participated in all phases of research and wrote the manuscript; MAS wrote the majority of the JAVA code; MAB contributed to software design and earlier code; RD gave intellectual contributions and revised the manuscript; SB contributed to the software code; MJM and PJS were involved in an earlier implementation of the software; BL gave intellectual contributions; PJV initiated the idea, gave intellectual contributions and revised the manuscript.
